# Toward First-in-Class Brain PET Tracers for Imaging TDP-43 Pathology

**DOI:** 10.1021/cbmi.6c00041

**Published:** 2026-03-27

**Authors:** Steven H. Liang, Run Wang, Chongzhao Ran, Neil Vasdev, Yinlong Li

**Affiliations:** † Department of Radiology and Imaging Sciences, 1371 Emory University, 1364 Clifton Road, Atlanta, Georgia 30322, United States; ‡ Wallace H. Coulter Department of Biomedical Engineering, Georgia Institute of Technology and Emory University, Atlanta, Georgia 30322, United States; § Insilico Medicine Shanghai Ltd, 2889 Jinke Road, Pudong New District, Shanghai 201203, China; ∥ Athinoula A. Martinos Center for Biomedical Imaging, Department of Radiology, Massachusetts General Hospital and Harvard Medical School, Boston, Massachusetts 02114, United States; ⊥ Azrieli Centre for Neuro-Radiochemistry, Brain Health Imaging Centre, Centre for Addiction and Mental Health (CAMH), Toronto M5T 1R8, Canada; # Department of Psychiatry, 7938 University of Toronto, Toronto M5T 1R8, Canada

Transactivation response element DNA binding Protein-43 kDa (TDP-43) is a defining pathological feature of amyotrophic lateral sclerosis (ALS), frontotemporal dementia (FTD),^1,2^ and limbic-predominant age-related TDP-43 encephalopathy (LATE).^3^ The accumulation of TDP-43 in the cytoplasm, forming inclusions, disrupts cellular function and contributes to neurodegeneration.^4,5^ TDP-43 has also been implicated in other neurodegenerative diseases, and has widespread impact on brain function.^6–8^ Despite its critical role in disease progression, the inability to visualize TDP-43 aggregates *in vivo* has hindered early diagnosis and effective disease monitoring. This limitation has significantly hampered efforts to understand disease mechanisms and evaluate treatment efficacy, particularly for conditions like ALS and FTD, where disease onset and progression vary widely.

TDP-43 proteinopathy is found post-mortem in approximately 45% of FTD cases, and is classified as FTLD-TDP to differentiate it from FTLD characterized by the accumulation of tau aggregates (FTLD-Tau) or FUS-related proteins.^2,9–11^ FTLD-TDP is further classified into four main subtypes (A, B, C, and D),^11–13^ each of which is associated with distinct clinical presentations and genetic mutations.^14–16^ Despite progress in understanding the pathology of these subtypes, the absence of sensitive *in vivo* biomarkers for visualizing TDP-43 aggregates remains a major barrier to advancing both diagnostic and therapeutic strategies for TDP-43-related diseases.

Unlike amyloid-β (Aβ)^17–19^ and tau,^20–22^ which are proteinopathy targets that have several positron emission tomography (PET) radiopharmaceuticals which provide critical data for unraveling the temporal evolution of Alzheimer’s disease (AD) pathology, TDP-43 presents additional unique imaging challenges due to its intracellular localization, structural variability, and co-occurrence with other proteinopathies.^23,24^ In addition to the difficulty of targeting intracellular aggregates, any TDP-43 tracer must also meet the rigorous demands of favorable pharmacokinetics. This includes high brain penetration, rapid washout to minimize background signal, and selectivity for TDP-43 aggregates, without cross-reactivity to other neurodegenerative deposits or other central nervous system targets.^25,26^ Consequently, PET radiotracers that target β-pleated sheet structures in TDP-43 proteinopathies including tau protein have been explored with minimal success in ALS.^27^ The development of a PET tracer that meets these stringent criteria is essential for advancing diagnostic and therapeutic strategies for neurodegenerative diseases within the TDP-43 spectrum of proteinopathies.

Toward this goal, a recent study by AC Immune reports the characterization of two first-in-class TDP-43 PET tracers,^28^ [^18^F]­ACI-19278 and [^18^F]­ACI-19626 (Figure 1). Both tracers are specifically designed to selectively target aggregated TDP-43 and optimized for brain penetration, metabolic stability, and selectivity over coexisting neurodegenerative protein aggregates, such as Aβ and Tau. While both compounds demonstrated moderate binding affinity for aggregated TDP-43 in vitro, ACI-19626 exhibited a higher target-to-background ratio and greater selectivity for TDP-43, positioned as the more promising candidate for in vivo imaging and was prioritized for first-in-human (FIH) evaluation in both healthy volunteers and patients with TDP-43 proteinopathies. These tracers represent a step forward to visualize TDP-43 aggregates *in vivo*, and hold the potential to play a key role in the early diagnosis and monitoring of TDP-43-associated diseases.

**Figure 1:**
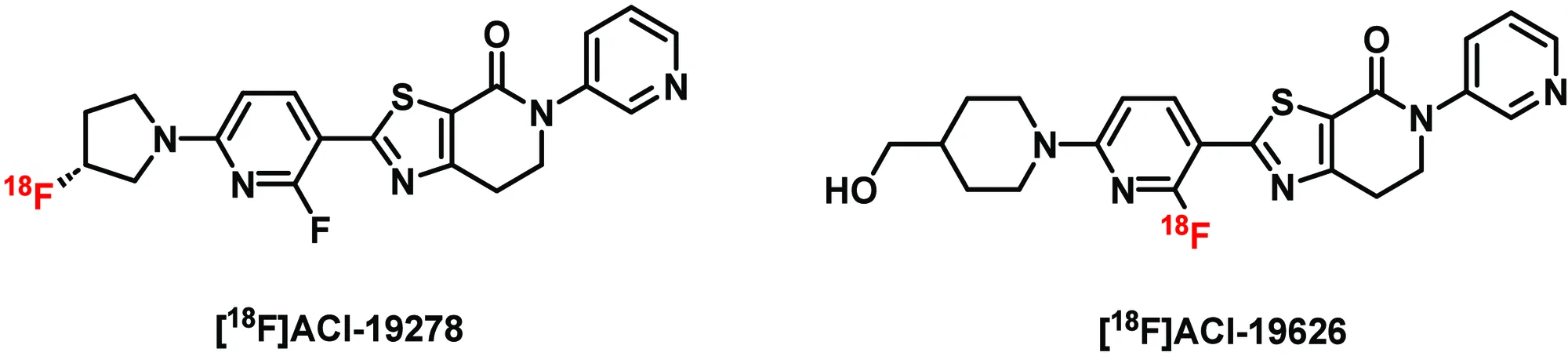
Chemical structures of [^18^F]­ACI-19278 and [^18^F]­ACI-19626.

In this study, [^3^H]­ACI-19278 and [^3^H]­ACI-19626 were assessed for binding to pathological TDP-43 aggregates using classical autoradiography (ARG) in brain samples from FTLD-TDP types A, B, and C, LATE-NC, ALS, and healthy controls. Both compounds had some affinity for aggregated TDP-43, with [^3^H]­ACI-19278 showing significant binding to FTLD-TDP type A, and [^3^H]­ACI-19626 exhibiting stronger binding to both FTLD-TDP types A and B (Figure 2A). Binding specificity was confirmed through displacement assays, which showed minimal nonspecific binding in control tissues. Saturation binding studies indicated that [^3^H]­ACI-19278 and [^3^H]­ACI-19626 have *K*_d_ values of 25 ± 25 nM and 18 ± 1 nM, respectively (Figure 2B), which may be suboptimal for TDP-43 aggregates, a low abundance target. Further binding studies with brain homogenates from FTLD-TDP types A and B verified the selectivity of both compounds for pathological TDP-43, with no binding observed in control tissues (Figure 2C). These results underscore the potential of [^3^H]­ACI-19278 and [^3^H]­ACI-19626 as scaffolds to develop imaging agents for TDP-43 aggregates, offering some specificity and minimal cross-reactivity with other protein deposits.

**Figure 2:**
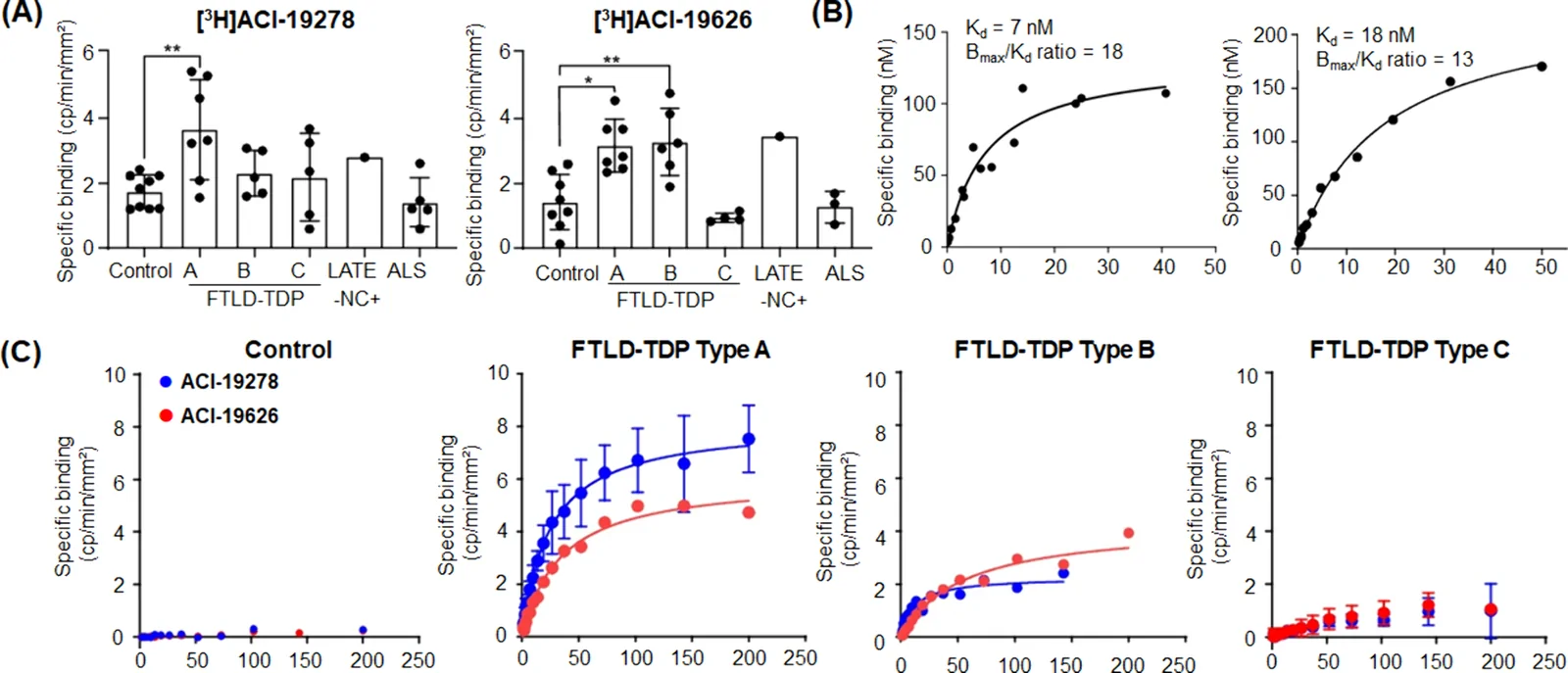
**Autoradiography assay of [** ^**3**^ **H]­ACI-19278 and [** ^**3**^ **H]­ACI-19626 to TDP-43 aggregates in ALS and FTD tissues, with minimal binding in control tissues**. (**A**) Binding specificity: ACI-19278 and ACI-19626. Data denotes mean ± SD (**B**) Saturation binding studies of [^3^H]­ACI-19278 and [^3^H]­ACI-19626 conducted on brain tissue from a donor with FTLD-TDP type A. (**C**) Saturation binding assays of [^3^H]­ACI-19278 and [^3^H]­ACI-19626 in brain homogenates from control, FTLD-TDP type A, B, and C cases. Data denotes mean ± SD for [^3^H]­ACI-19626 on type A and for both tracers on type C. The data was adapted from ref.^28^ Copyright 2025 Springer Nature.

To better understand the binding specificity of [^3^H]­ACI-19278 and [^3^H]­ACI-19626 for aggregated TDP-43, high-resolution ARG studies were performed.^29^ Both tracers demonstrated selective binding to aggregated TDP-43 in FTLD-TDP types A and B, LATE-NC, and ALS tissues, with no binding observed in control tissues (Figure 3A). Surface plasmon resonance studies confirmed the moderate affinity for aggregated TDP-43 (*K*_d_ values: 60 ± 9 nM for ACI-19278 and 80 ± 26 nM for ACI-19626) with no binding to soluble TDP-43 (Figure 3B). Furthermore, [^3^H]­ACI-19278 showed modest colocalization (∼50%) with fibrillar TDP-43 in cellular models, for aggregated over physiological TDP-43 (Figure 3C). Importantly, ACI-19278 did not interfere with TDP-43’s physiological function, as shown by a CFTR exon 9 splicing assay (Figure 3D), confirming its specificity for pathological TDP-43 aggregates without disrupting normal cellular processes. Collectively, these results show that both compounds have some potential to bind selectively to aggregated TDP-43, with no disruption to its normal cellular function, thus making their scaffold a reasonable starting point for developing imaging agents for TDP-43 pathology in neurodegenerative diseases.

**Figure 3:**
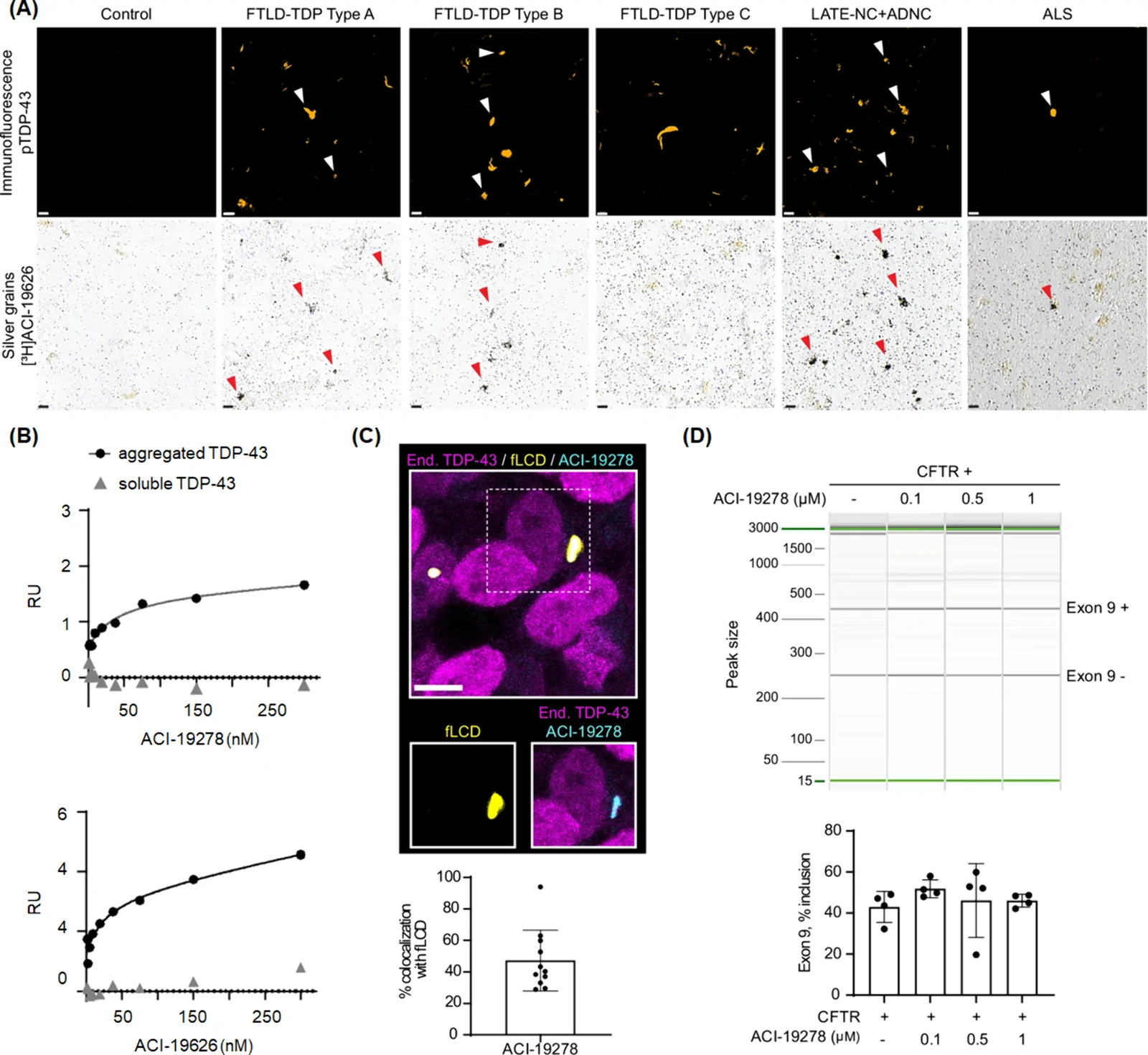
**Selective recognition of aggregated TDP-43 by ACI-19278 and ACI-19626. (A)** Autoradiography assays of [^3^H]­ACI-19626 (20 nM) in tissue sections from FTLD-TDP type A, B and C, LATE-NC + ADNC, ALS and a control group, with phospho-TDP-43 pS409/410 (pTDP-43) antibody staining (white arrowheads). The binding of silver grains to pTDP-43 inclusions (red arrowheads) indicates tracer colocalization. Scale bar = 10 μm. (B) Surface plasmon resonance studies assays to assess the binding affinity of ACI-19278 and ACI-19626 for aggregated TDP-43 and soluble TDP-43. Data shown from one of four independent experiments. (C) ACI-19278 binds efficiently to TDP-43 fibrils, excluding nuclear TDP-43. Immunofluorescence of SH-SY5Y cells treated with TDP-43 fibrils and ACI-19278. Quantification of colocalization shown. Data denotes mean ± SD from two independent experiments. (D) ACI-19278 does not affect TDP-43 function. RT-PCR analysis of CFTR exon 9 inclusion in SH-SY5Y cells with ACI-19278 (0.1–1 μM). Data denotes mean ± SD from four experiments. The data was adapted from ref.^28^ Copyright 2025 Springer Nature.

Having demonstrated strong on-target binding to TDP-43, the binding specificity of [^3^H]­ACI-19278 and [^3^H]­ACI-19626 for aggregated TDP-43 over common brain amyloid copathologies was further evaluated in frontal cortex sections from an AD brain devoid of TDP-43 pathology. Radiolabeled reference tracers for Aβ and Tau showed strong, displaceable binding, correlating with the distribution of Aβ plaques and Tau aggregates, as confirmed by immunofluorescence (Figure 4A, top panel). In contrast, [^3^H]­ACI-19278 and [^3^H]­ACI-19626 exhibited minimal to no binding, indicating preferential binding to TDP-43 aggregates over Aβ and Tau (Figure 4A). This selectivity was further confirmed by high-resolution ARG combined with immunolabeling of phosphorylated Tau (pTau, Figure 4B, C), where both tracers exhibited no binding to Tau aggregates in FTLD-Tau tissue, while the Tau-specific tracer [^3^H]­PI-2620 bound strongly to tau tangles.^30^ Moreover, as shown in Figure 4D, neither [^3^H]­ACI-19278 nor [^3^H]­ACI-19626 exhibited binding to α-synuclein aggregates in PD brain tissue, further reinforcing their selectivity for TDP-43 over Aβ, Tau, and α-synuclein. Collectively, these findings confirm that [^3^H]­ACI-19278 and [^3^H]­ACI-19626 bind with some selectively to aggregated TDP-43, positioning them as promising starting points for developing new chemical entities for imaging TDP-43 pathology in neurodegenerative diseases.

**Figure 4:**
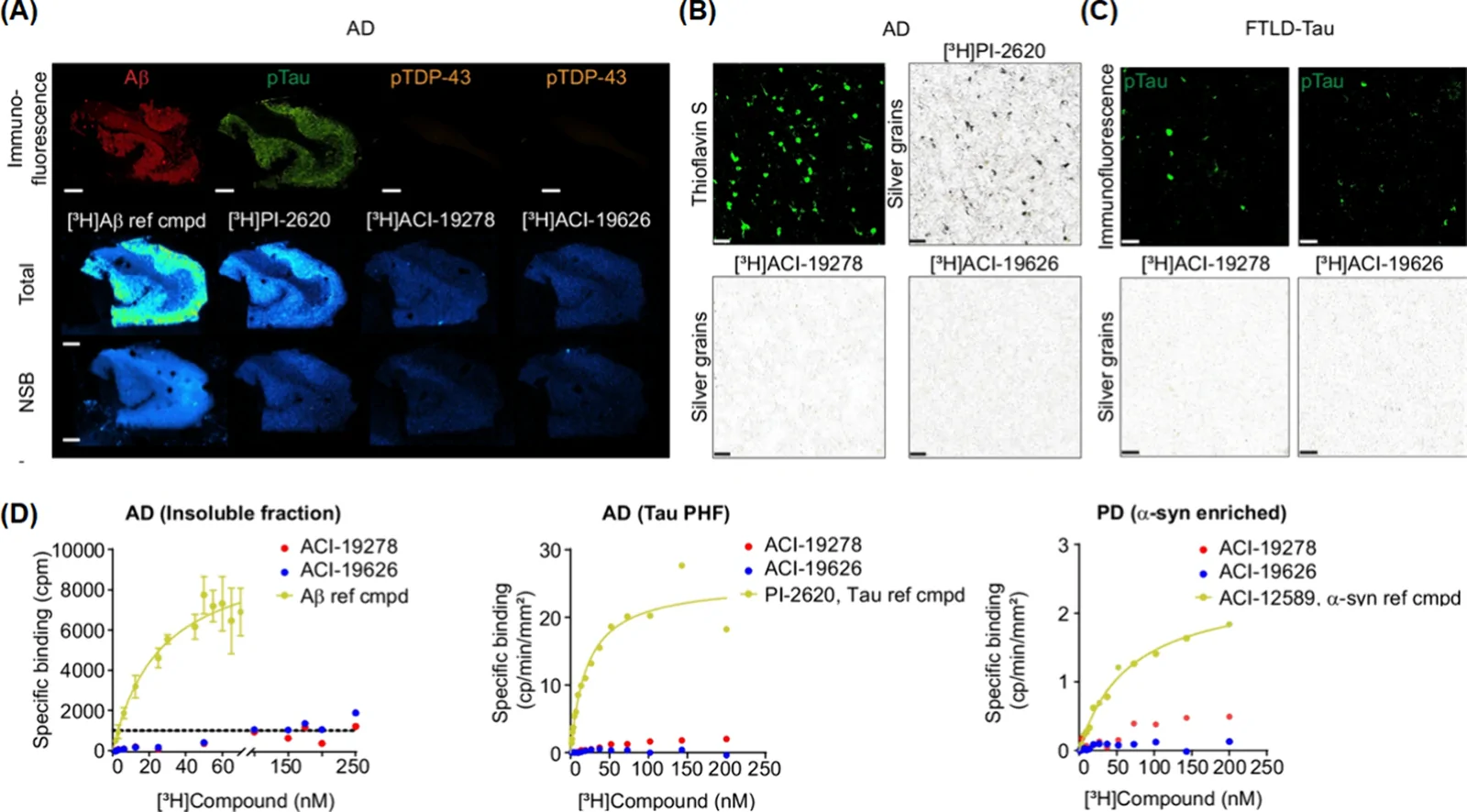
**Selectivity of ACI-19278 and ACI-19626 for TDP-43 aggregates over common copathologies. (A)** Autoradiography in Alzheimer’s disease tissue showing binding of the tracers to amyloid and Tau aggregates, with minimal nonspecific binding. Immunolabeling for phospho-TDP-43, phospho-Tau or Aβ in adjacent tissue sections. Scale bar, 2 mm. (B) High-resolution imaging in the entorhinal cortex of an AD patient, comparing tracer binding with Thioflavin S staining to highlight amyloid plaques. Scale bar, 50 μm. (C) High-resolution autoradiography in FTLD-Tau tissue, showing selective binding with phospho-Tau immunolabeling. Scale bar, 50 μm. (D) Saturation binding studies of [^3^H]­ACI-19278 and [^3^H]­ACI-19626 in brain homogenates from AD, PD, and control tissues. Reference ligands for Aβ, Tau, and α-synuclein were also tested. Data denotes mean ± SD of at least two independent experiments for each tracer, with representative data shown for [^3^H]­Aβ ref cmpd in AD homogenates. The data was adapted from ref.^28^ Copyright 2025 Springer Nature.

The pharmacokinetic profiles of [^18^F]­ACI-19278 and [^18^F]­ACI-19626 were evaluated in rhesus macaques through dynamic PET scans (0–180 min). Both compounds exhibited rapid brain uptake, indicating efficient CNS penetration (Figure 5). As shown in Figure 5B, [^18^F]­ACI-19278 reached peak brain uptake at 5.5 min, with a standardized uptake value (SUV) of 2.9, while [^18^F]­ACI-19626 peaked at 4.5 min with an SUV of 1.1, reflecting the higher retention of [^18^F]­ACI-19278 in the brain. This difference in retention is consistent with [^18^F]­ACI-19278’s lower lipophilicity compared to ACI-19626. Time-activity curves (TACs) showed that both tracers exhibited rapid washout, with [^18^F]­ACI-19278 demonstrating a brain concentration ratio between peak and 60 min of 5.4, compared to 15.5 for [^18^F]­ACI-19626 (Figure 5C). These pharmacokinetic findings suggest that both tracers have favorable profiles for PET imaging, with potential for high-quality *in vivo* visualization of TDP-43 aggregates, however, further characterization will be required to confirm that the tracers are indeed imaging TDP-43 in vivo.

**Figure 5:**
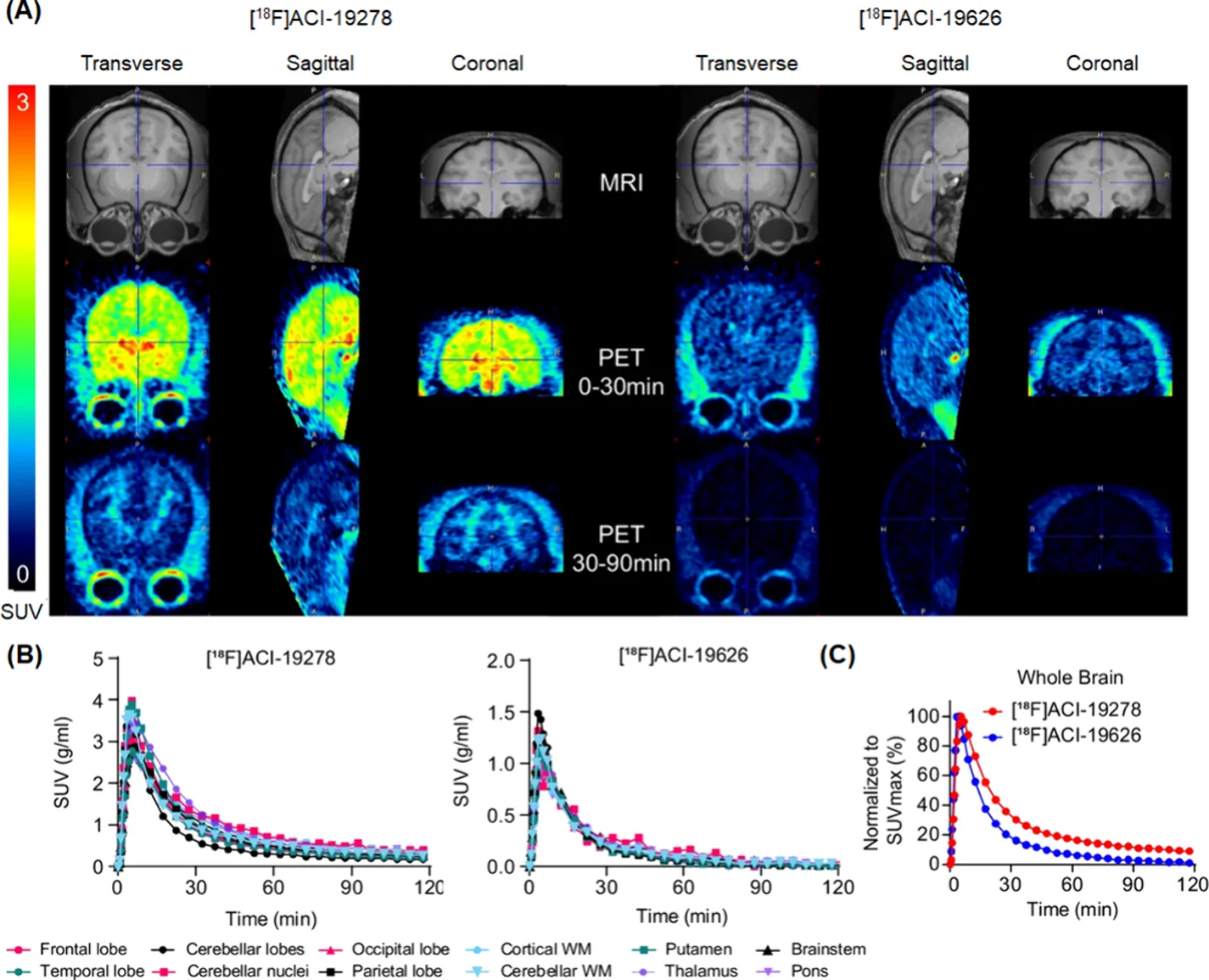
**Pharmacokinetic profiles of [** ^**18**^ **F]­ACI-19278 and [** ^**18**^ **F]­ACI-19626. (A)** MRI and PET images in transverse, sagittal, and coronal views illustrating the determination of SUV averaged over 0–30 min (middle row) and 30–90 min postinjection (bottom row). (B) Time-activity curves derived from SUV quantification for [^18^F]­ACI-19278 or [^18^F]­ACI-19626 in different brain regions. Data from one experiment. (C) The TACs comparison for [^18^F]­ACI-19278 and [^18^F]­ACI-19626 averaged across the whole brain, reported as a percentage of the highest uptake (SU*V*_max_) for each tracer. The data was adapted from ref.^28^ Copyright 2025 Springer Nature.

## Concluding Remarks

The performance of [^18^F]­ACI-19278 and [^18^F]­ACI-19626 paves the way for their chemical scaffolds to be optimized for more potent radiotracers for TDP-43, that can ultimately be integrated into clinical research, enabling noninvasive, real-time visualization of TDP-43 aggregates in patients with neurodegenerative diseases. A notable strength is the multidisciplinary, cross-validated workflow that anticipates the heterogeneity of TDP-43 proteinopathies and the confounds introduced by mixed pathologies in aging. Future efforts can focus on optimizing their potency, selectivity, pharmacokinetics, enhancing bioavailability, and validating them in human trials to ensure their clinical applicability. At the same time, translation will require broader sampling across brain regions and underrepresented genetic/pathological subtypes (including FTLD-TDP subtype E), and careful consideration of subtype-specific biology highlighted by absent binding in FTLD-TDP C. TDP-43 tracers hold the potential for early diagnosis, disease monitoring, and evaluating therapeutic efficacy, and remain an active goal for understanding neurodegenerative diseases. Moreover, the principles behind their development could inform the design of molecular imaging agents targeting other intracellular protein aggregates, expanding their use across various neurodegenerative conditions. Because TDP-43 inclusions can be relatively sparse and tightly coupled to stage-dependent neurodegeneration and regional atrophy, clinical readout strategies will likely need to incorporate disease stage, phenotype, and cortical volumes when defining diagnostic thresholds. As research progresses, combining PET imaging with emerging TDP-43-modulating therapies may lead to theranostic applications that not only visualize but also intervene in disease mechanisms, thus accelerating drug development and improving patient outcomes.^31,32^ The ability to monitor TDP-43 *in vivo* could revolutionize both clinical diagnostics and therapeutic strategies for these debilitating diseases.

## Abbreviations

ALS: Amyotrophic lateral sclerosis
AD: Alzheimer’s disease
Aβ: Amyloid-beta
C9orf72: Chromosome 9 open reading frame 72
CTE: Chronic traumatic encephalopathy
DLB: Dementia with Lewy bodies
FTD: Frontotemporal dementia
FTLD-TDP: Frontotemporal lobar degeneration with TDP-43 proteinopathy
FTLD-Tau: Frontotemporal lobar degeneration with Tau proteinopathy
FTLD-FUS/FET: Frontotemporal lobar degeneration with FUS-Ewing sarcoma-TAF15 family proteinopathy
LATE: Limbic-predominant age-related TDP-43 encephalopathy
pTau: Phosphorylated Tau
svPPA: Semantic variant primary progressive aphasia
naPPA: Nonfluent agrammatic primary progressive aphasia
bvFTD: Behavioral variant frontotemporal dementia
TDP-43: Transactive response DNA-binding protein 43
PET: Positron emission tomography
SUV: Standardized uptake value
FIH: First-in-human
